# Apoptosis induced by a HIPK2 full-length-specific siRNA is due to off-target effects rather than prevalence of HIPK2-Δe8 isoform

**DOI:** 10.18632/oncotarget.6423

**Published:** 2015-11-28

**Authors:** Giuliana Di Rocco, Alessandra Verdina, Veronica Gatti, Ilaria Virdia, Gabriele Toietta, Matilde Todaro, Giorgio Stassi, Silvia Soddu

**Affiliations:** ^1^ Department of Research, Advanced Diagnostics, and Technological Innovation, Regina Elena National Cancer Institute, Rome, Italy; ^2^ Department of Surgical and Oncological Sciences, Cellular and Molecular Pathophysiology Laboratory, University of Palermo, Palermo, Italy

**Keywords:** HIPK2, alternative splicing isoforms, colorectal cancer, siRNA therapeutic application, off-target effects

## Abstract

Small interfering RNAs (siRNAs) are widely used to study gene function and extensively exploited for their potential therapeutic applications. HIPK2 is an evolutionary conserved kinase that binds and phosphorylates several proteins directly or indirectly related to apoptosis. Recently, an alternatively spliced isoform skipping 81 nucleotides of exon 8 (Hipk2-Δe8) has been described. Selective depletion of Hipk2 full-length (Hipk2-FL) with a specific siRNA that spares the Hipk2-Δe8 isoform has been shown to strongly induce apoptosis, suggesting an unpredicted dominant-negative effect of Hipk2-FL over the Δe8 isoform. From this observation, we sought to take advantage and assessed the therapeutic potential of generating Hipk2 isoform unbalance in tumor-initiating cells derived from colorectal cancer patients. Strong reduction of cell viability was induced *in vitro* and *in vivo* by the originally described exon 8-specific siRNA, supporting a potential therapeutic application. However, validation analyses performed with additional exon8-specific siRNAs with different stabilities showed that all exon8-targeting siRNAs can induce comparable Hipk2 isoform unbalance but only the originally reported e8-siRNA promotes cell death. These data show that loss of viability does not depend on the prevalence of Hipk2-Δe8 isoform but it is rather due to microRNA-like off-target effects.

## INTRODUCTION

Colorectal cancer (CRC) is the third most common cancer globally with more than 1.2 million new cases diagnosed globally and roughly 600,000 deaths occurring every year [1]. The prognosis of CRC patients has improved in the past decades with a 5-year relative survival greater than 90% in patients with stage I disease. These major advances have been achieved through multidisciplinary approaches, ranging from the improved screening to identify individuals at early stages to the development of targeted therapies [2]. Yet, there is still approximately a 10% 5-year relative survival rate in patients with stage IV disease [2] calling for further advances in prognostic and predictive markers and molecular targeted treatments.

RNA interference (RNAi) is a sequence-specific, RNA-dependent gene-silencing process occurring in many eukaryotes, including humans [3]. In mammalian cells, RNAi can be mediated by short RNAs of about 20 nucleotides that include endogenous microRNAs and exogenous short hairpin RNAs and synthetic siRNAs [4]. At the functional level, RNAi contributes to different physiological or pathological processes, including regulation of gene expression during differentiation and development, deregulation of oncogenes and tumor suppressor genes in tumorigenesis, cell defense against parasitic nucleotide sequences such as viruses and transposons [5, 6]. Given its selectivity in gene silencing, RNAi has become a potent and valuable research tool to study gene function both in cell culture and in living organisms [7]. Translation of this strategy into clinical applications for disease treatment is being extensively exploited and a few siRNA-based specific gene silencing have entered clinical trials [8–11]. Indeed, significant improvement of delivery, bioavailability, and safety for the siRNA therapeutics has strongly stimulated the preclinical evaluation of a large body of siRNA targets with potential drug activity [12, 13]. This makes recognition of siRNA off-target effects a key aspect for both target identification and therapeutic applications [14].

Homeodomain-interacting protein kinase 2 (HIPK2) is a tyrosine-regulated serine/threonine kinase [15, 16] that phosphorylates a large body of proteins belonging to different networks, including transcriptional regulators, chromatin modifiers, signal transducers, and E3 components of SUMO ligases [17]. Data supporting a role for HIPK2 as tumor suppressor have been obtained in mice where the *hipk2* gene behaves as a haploinsufficient tumor suppressor in γ-irradiation-induced thymic lymphomagenesis [18], and in humans, where HIPK2 has been found inactivated by different mechanisms in different cancer types [19–21]. In response to genotoxic damage, HIPK2 stimulates p53–depended and –independent cell cycle arrest and apoptosis [22–24] and specific reduction of HIPK2 expression by anti-sense oligonucleotides or RNAi was shown to impair apoptosis and induce resistance to different anticancer treatments [25, 26]. In a recent study regarding the role of the serine/arginine-rich splicing factor 3 in colon cancer cells [27], Kurokawa and colleagues uncovered the existence of an alternative splicing form of HIPK2, the Hipk2-Δe8 isoform, which is generated by the skipping of 81 5′-nucleotides from exon 8. Interestingly, by employing a siRNA that hybridizes with the skipped exon 8 region and selectively depletes Hipk2-FL (here referred to as e8-siRNA#1), the authors observed a strong induction of apoptosis in both p53-proficient and -defective CRC cells [27]. Based on these data, the authors proposed that prevalence of Hipk2-Δe8 isoform expression over the FL one is a potent inducer of apoptosis [27]. Besides the mechanistic implications in the biological activity of HIPK2, this observation opens up to a stimulating opportunity to test the therapeutic efficacy of Hipk2-FL targeting siRNAs in human CRCs.

Here, we investigated the therapeutic potential of the imbalance between Hipk2-Δe8 and Hipk2-FL isoforms induced by e8-siRNA#1 in a series of CRC cells including patient-derived tumor-initiating cells (*i.e.*, Cancer Stem Cells – CSCs) [28]. We confirm the loss of viability *in vitro* by e8-siRNA#1 (Hipk2 siRNA#2 in Kurokawa et al.) and further show its ability to efficiently reduce the growth of CSC-derived tumor xenografts *in vivo*. However, target validation experiments performed by employing multiple individual siRNAs targeting the same skipped exon 8 region of Hipk2 showed a comparable unbalance between Hipk2-Δe8 and Hipk2-FL isoforms but no sign of cell death, demonstrating that loss of viability is due to off-target effects.

## RESULTS

**Figure 1:**
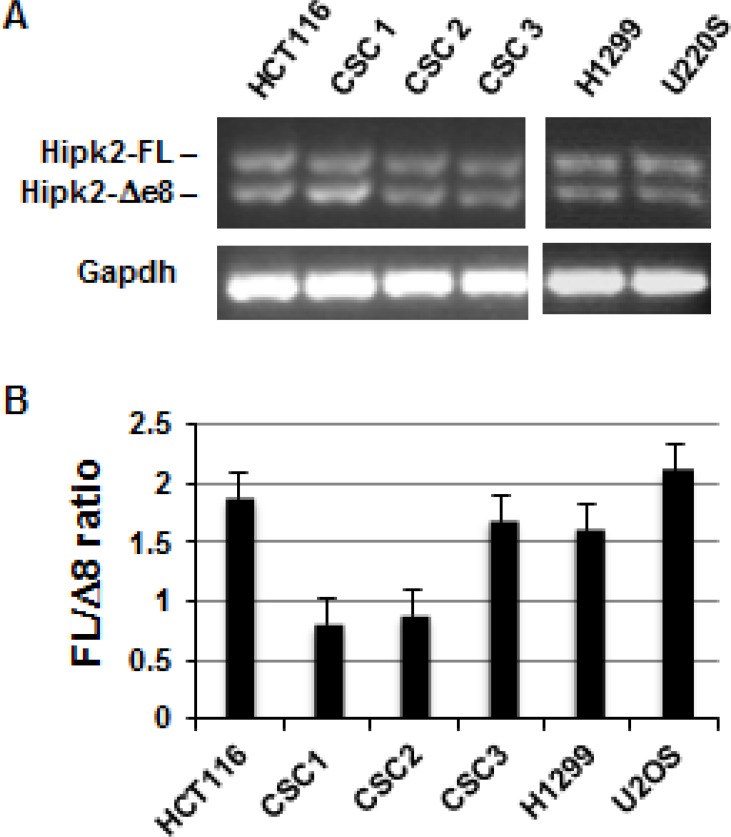
Both Hipk2 RNA isoforms are expressed in tumor cell lines and patient-derived colon CSCs (**A**) Expression of Hipk-2-FL and Hipk2-Δe8 isoform were measured by RT-PCR using a primer set which amplifies the alternatively spliced region of exon 8. Gapdh was used as loading control. (**B**) Quantitative Real Time-PCR analysis with isoform-specific primers was used to determine the relative ratio of Hipk2-FL and Hipk2-Δe8 mRNA expression.

To explore the potential therapeutic activity of Hipk2-FL specific targeting by siRNA in CRCs, we first evaluated the expression of the two Hipk2 isoforms, Hipk2-FL and Hipk2-Δe8, in three patient-derived colorectal CSCs (CSC1, CSC2, and CSC3) [29]. In this and in further experiments, we included the HCT-116 cells for a direct comparison with the already reported results [27] and the p53-null H1299 cells and the wild-type p53-carrying U2OS cells to verify the generality and the p53-independency of the cell death induced by Hipk2 isoform-unbalance. Due to the absence of HIPK2 isoform-specific antibodies [27], RT-PCR was performed with primers external to the spliced region of exon 8 to check for Hipk2 isoform expression. Both Hipk2 isoforms were detected in all the analyzed cells (Figure 1A). In addition, Real Time quantitative PCR analysis showed that the relative ratio between the FL and Δe8 Hipk2 isoforms varies between 0.7 and 2.1 in tested cells (Figure 1B). This result provides the possibility of triggering an e8-siRNA#1-induced imbalance of the Hipk2 isoforms and verify its therapeutic efficacy at the preclinical level.

### e8-siRNA#1 induces cells death in CRC cells

**Figure 2:**
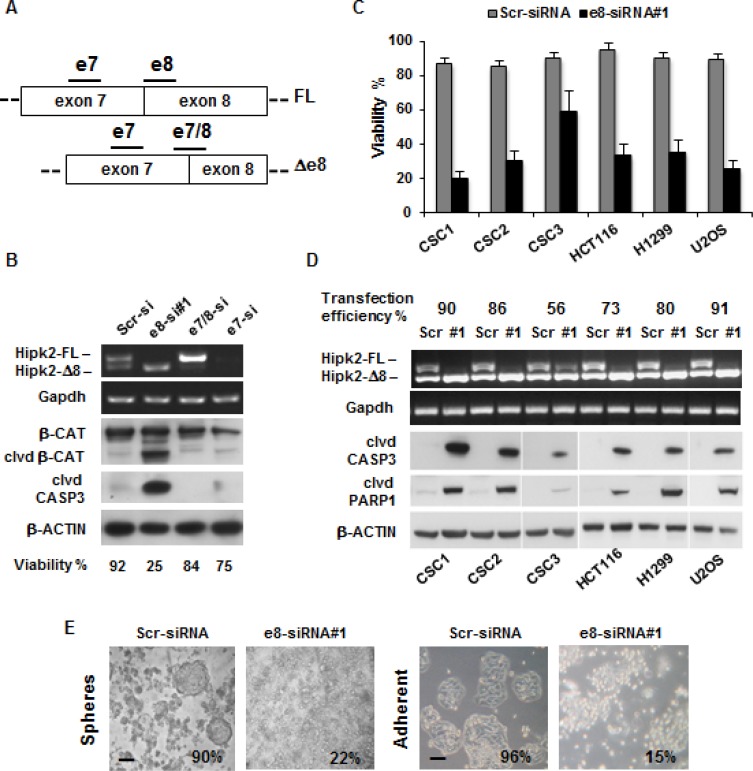
The e8-siRNA#1 targeting the Hipk2-FL isoform induces cell death in tumor cells and colon CSCs (**A**) Schematic representation of Hipk2 isoforms in the alternative spliced region. Bars indicate the relative annealing position of siRNAs targeting both isoforms (e7-siRNA), the FL isoform (e8-siRNA#1) or the Δ8 isoform (e7/8-siRNA), respectively. (**B**) CSC1 were transfected with the indicated siRNAs and collected after 72 hrs. Cells from the same plate were counted to assess viability and analyzed by both RT-PCR, to check for specific Hipk2 isoform depletion and WB, to detect apoptosis. Cleaved CASPASE 3 (clvd CASP3) and β-CATENIN (clvd β-CAT) were used as apoptotic markers, while Gapdh and β-ACTIN were used as loading controls. (**C**) Cell were transfected with e8-siRNA#1 or its scrambled version (Scr-siRNA) and collected after 72 hrs. Cell viability was determined by Trypan-blue exclusion test. Values are expressed as means ± standard deviation (S.D.) (*n* = 3). (**D**) Cell were transfected as in C and analyzed by RT-PCR, to check for specific Hipk2 isoform depletion and by WB, to detect apoptosis. Cleaved CASPASE 3 (clvd CASP3) and PARP-1 (clvd PARP1) were used as apoptotic markers, Gapdh and β-ACTIN were used as loading controls. A fluorescent oligo was co-transfected with the control siRNA to check for transfection efficiency. (**E**) CSC1 cells were cultured as spheroids in the absence of serum in low-adherence plates (upper panels) or let attach on standard tissue culture plates in the presence of serum (bottom panels) and compared for their sensibility to e8-siRNA apoptotic effect. Pictures were taken after three days of treatment. The percentage of viability is indicated at the bottom of each panel. Bars, 100 μm

Selective depletion of Hipk2-FL by e8-siRNA#1 was shown to induce apoptosis in p53-proficient and p53–defective cells [27]. To assess whether this effect can be extended to patient-derived CSCs and eventually employed as targeted drugs in CRCs, CSCs were transfected with each of the following Hipk2-specific siRNAs that hybridize with three different regions: a siRNA targeting both isoforms on exon 7 (e7-siRNA), a siRNA targeting the skipped exon 8 present only in the Hipk2-FL isoform (e8-siRNA#1), and a siRNA targeting the junction between exon 7 and exon 8 (e7/8-siRNA) present only in the Hipk2-Δe8 isoform (Figure 2A). A siRNA with a scrambled sequence of e8-siRNA#1 was used as control. At 72 hrs post-transfection, cells were collected and analyzed by RT-PCR for Hipk2 isoform expression and by Western blotting (WB) for apoptotic markers (*i.e*., cleaved forms of Caspase 3 and β-Catenin) [30]. Representative results obtained with CSC1 cells are reported in Figure 2B and show that the three Hipk2-specific siRNAs efficiently deplete Hipk2 isoforms as predicted from their respective annealing sequences (Figure 2A–2B). Consistently with the data reported by Kurokawa and colleagues [27], only the e8-siRNA#1 that selectively depletes the Hipk2-FL isoform was associated with strong loss of viability and the appearance of apoptotic markers (Figure 2B). Loss of viability induced by e8-siRNA#1 transfection and the induction of apoptotic markers (*i.e*., cleaved forms of Caspase 3 and PARP1) were confirmed in all the cells employed and the amount of cell death was proportional to the transfection efficiency (Figure 2C–2D), making unlikely a cell-specific response.

Considering that the patient-derived CSCs are usually maintained as spheres in strictly defined, serum-free medium [28], we compared the amount of cell death induced by the e8-siRNA#1-treatment in CSCs cultured as spheres in low-adherence plates without serum, or maintained in adherent conditions and in the presence of fetal bovine serum (FBS). No significant differences were observed in the loss of viability in the two culturing conditions (Figure 2E), indicating that in these CRC cells the e8-siRNA#1-induced death is both substrate and growth factor independent.

### e8-siRNA#1 reduces tumor growth in CSC-derived tumor xenograft

**Figure 3:**
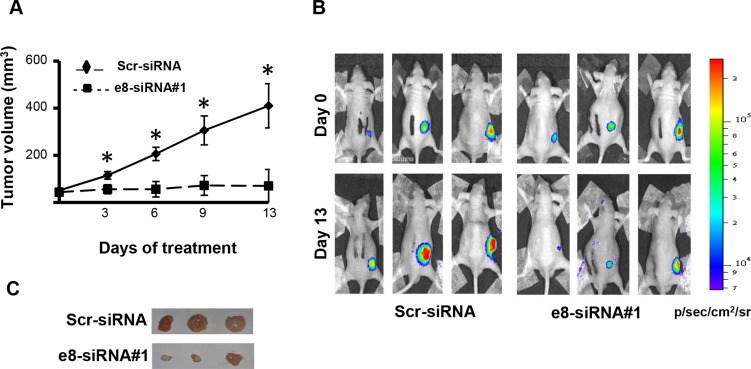
Treatment with e8-siRNA#1 inhibits tumor growth *in vivo* Patient derived CSC1 cells stably transfected with a luciferase reporter vector were inoculated into athymic mice. Twice a week mice were intratumorally treated with e8-siRNA (*n* = 6). A scrambled version of e8-siRNA (Scr-siRNA) was used as control (*n* = 6). (**A**) Tumor volume in nude mice treated with e8-siRNA or with Scr-siRNA. Xenograft growth was monitored and showed as mean ± S.D. *p < 0.01. (**B**) Bioluminescence imaging of three representative mice for each group at 0 and 13 days of treatment. Luciferase imaging of mice whole body was performed twice a week. (**C**) Comparison of tumors explanted from Scr-siRNA and e8-siRNA#1 treated mice fifteen days after the first treatment. Three representative tumors for each group are shown.

To begin evaluating the possible therapeutic efficacy of e8-siRNA#1-treatment *in vivo*, patient-derived CSC1 cells were stably transfected with a luciferase reporter vector to allow *in vivo* imaging [31] and then injected subcutaneously into nude mice. Seven days post-injection, when tumors were palpable and visible by bioluminescence imaging, mice were intratumorally injected with the e8-siRNA#1 using RNAi-Max Lipofectamine as a delivery vehicle. A scrambled sequence of e8-siRNA#1 (Scr-siRNA) was used as control. Compared to control, e8-siRNA#1 treatment strongly reduced tumor growth measured by both tumor calibration (Figure 3A) and luciferase intensity (Figure 3B). This effect was confirmed by direct explanted tumors comparison fifteen days after the first treatment, showing a reduction in size and decreased vascularization in the e8-siRNA#1-treated CSC1 tumor xenografts (Figure 3C). These results emphasize that at least the direct intratumor injection of the e8-siRNA#1 can be therapeutically effective in a tumor xenograft model, calling for further preclinical tests.

### Target validation reveals a clear off-target cell death activity of the e8-siRNA#1

**Figure 4:**
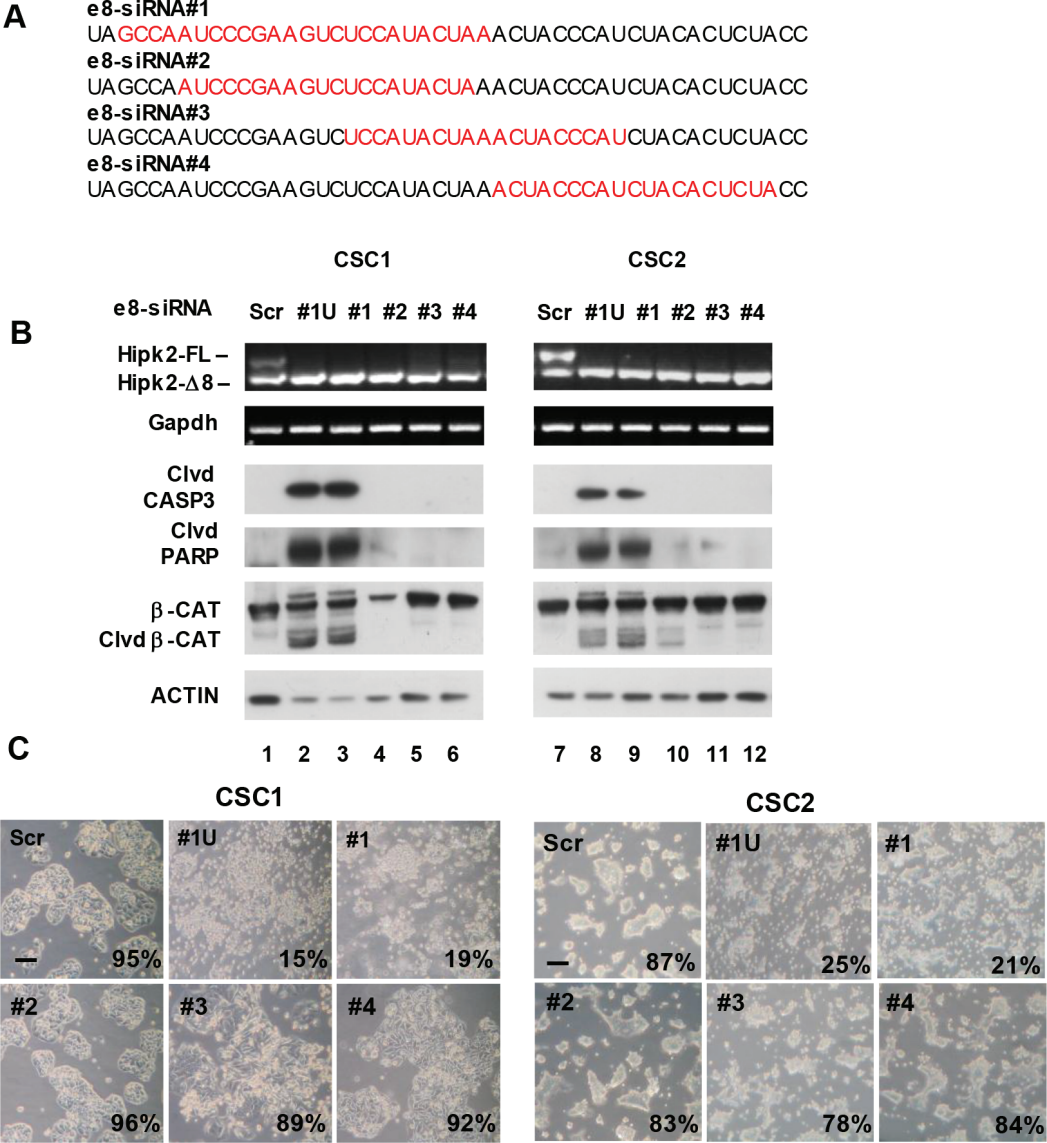
The use of different siRNAs targeting the same exon 8 region reveals that Hipk2-FL depletion is not sufficient to prompt apoptosis (**A**) Four different siRNAs homologous to a short region of Hipk2 exon 8 present only in the FL isoform were used for target validation. The sequence of each e8-siRNA is highlighted in red. (**B**) Two CSC lines were transfected with the indicated siRNAs. All siRNAs were used as Stealth RNAi from Invitrogen, with the exception of e8-siRNA#1U, which is a standard unmodified version of e8-siRNA#1. Cells were collected after 72 hrs, counted to assess viability and analyzed by both RT-PCR and WB. Cleaved CASPASE 3 (clvd CASP3) and β-CATENIN (clvd β-CAT) and PARP1 (Clvd PARP) were used as apoptotic markers. (**C**) Light microscopy pictures of transfected cells taken 96 hrs post-transfection reveal a clearly different phenotypic aspect in cells treated with different siRNA targeting the same RNA region. The percentage of viable cells is reported at the right bottom of each panel. Bars, 100 μm.

**Figure 5:**
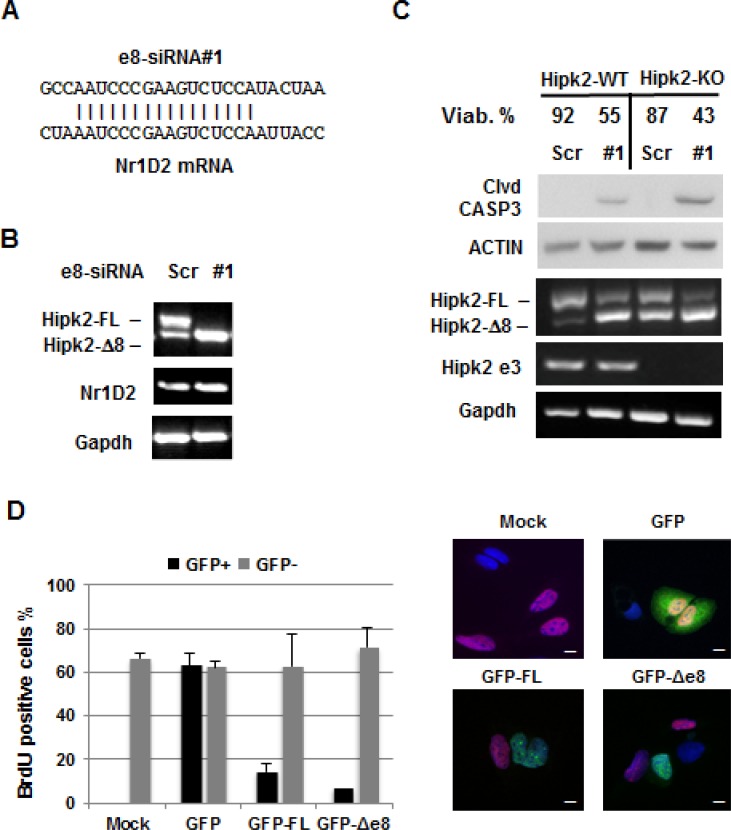
Evaluation of the e8-siRNA#1 off-target effect (**A**) Sequence alignment between Hipk2 e8siRNA#1 and Nr1D2 in the homologous region. (**B**) CSC1 cells were transfected with the indicated siRNAs. At 96 hrs post-transfection, Hipk2-FL and Nr1D2 expression were monitored by RT-PCR. (**C**) Hap-1 parental (Hipk2-WT) and Hipk2-KO cells were transfected with e8-siRNA#1 or its scrambled version (Scr-siRNA) and collected after 72 hrs. Cells were counted to assess viability and analyzed by both RT-PCR and WB. Cleaved CASPASE 3 (clvd CASP3) was used as apoptotic marker. Deletion of exon 3 in Hipk2-KO cells was confirmed by specific primers (Hipk2 e3). (**D**) U2OS cells were transfected with GFP-tagged constructs for HIPK2-FL (GFP-FL) and HIPK2-Δe8 (GFP-Δ8) isoforms. HIPK2 expressing cells were identified by GFP expression. BrdU positive, proliferating cells, were detected by immunofluorescence with an anti-BrdU antibody. Representative fluorescent images are reported in the right panels. Bars, 10 μm.

The use of multiple individual siRNAs targeting the same gene is considered a straightforward approach for target validation [32]. Thus, before proceeding to more demanding preclinical experiments, we performed further target validation tests to increase our confidence in the validity and specificity of the cell death induced by e8-siRNA#1-treatment. To this aim, three more Hipk2-FL-specific siRNAs (*i.e*., e8-siRNA#2, #3 and #4) that also hybridize within the skipped exon 8 region (Figure 4A) were produced and tested on CSC1 and CSC2 cells for stimulation of both Hipk2 isoform imbalance and loss of viability. In agreement with the data reported [27], each of the four employed siRNAs comparably repressed the Hipk2-FL expression, sparing the Hipk2-Δe8 isoform (Figure 4B, upper panels). Disappointingly, only the original e8-siRNA#1 was able to induce cell death, as shown by evaluation of Caspase 3, PARP1 and β-Catenin cleavage (Figure 4B, lower panels) and cell viability (Figure 4C), clearly indicating an off-target apoptotic activity of this specific siRNA. Thus, we first verified a possible lack of specificity due to mRNA degradation mediated by partial sequence complementation. An *in silico* search for annealing sequences of the e8-siRNA#1 with nonspecific genes showed a 64% complementation with the *NR1D2* (nuclear receptor subfamily 1, group D, member 2) gene (Figure 5A). NR1D2 is a DNA-binding transcriptional regulator involved in metabolic functions, inflammatory response, and circadian rhythm [33]. However, when we assessed the *NR1D2* mRNA levels after e8-siRNA#1 transfection, no reduction of expression was observed (Figure 5B).

Next, we evaluated the possibility that the high stability of the chemically modified e8-siRNAs used by Kurokawa and by our group (see Materials and Methods) might be responsible for the off-target-induced cell death. CSC1 and CSC2 cells were transfected with the chemically modified Stealth RNAi (e8-siRNA#1) or with the corresponding, unmodified siRNA (#1U). As shown in Figure 4B (lanes 2, 3 and 8, 9) and Figure 4C, comparable results were obtained with the two siRNA preparations in both cell populations ruling out a major effect due to high siRNA stability.

Eventually, we tested whether the pro-apoptotic activity of HIPK2 is required for the e8-siRNA#1-induced cell death. To this aim, we employed the parental Hap-1 cells (Hipk2-WT) and their Hipk2-KO derivatives, obtained by *Hipk2* exon 3 deletion through the CRISPR-Cas9 technology (see Materials and Methods). As shown in Figure 5C, e8-siRNA#1 similarly reduced cell viability in both cell types, indicating that the presence of neither of Hipk2 isoform is necessary for e8-siRNA#1-induced cell death. As further control, we generated an unbalance between the Hipk2 isoforms by their overexpression rather than silencing. As shown in Figure 5D, the GFP-tagged version of both HIPK2-FL and HIPK2-Δe8 isoforms significantly reduced BrdU incorporation when expressed in U2OS cells, supporting a similarity of action between the two isoforms.

Taken together, these results show that loss of viability induced by the e8-siRNA#1 does not depend on the unbalance between Hipk2 isoforms but it is rather caused by off-target activity of this specific siRNA sequence.

### The e8-siRNA#1 off-target activity has no therapeutic window

**Figure 6:**
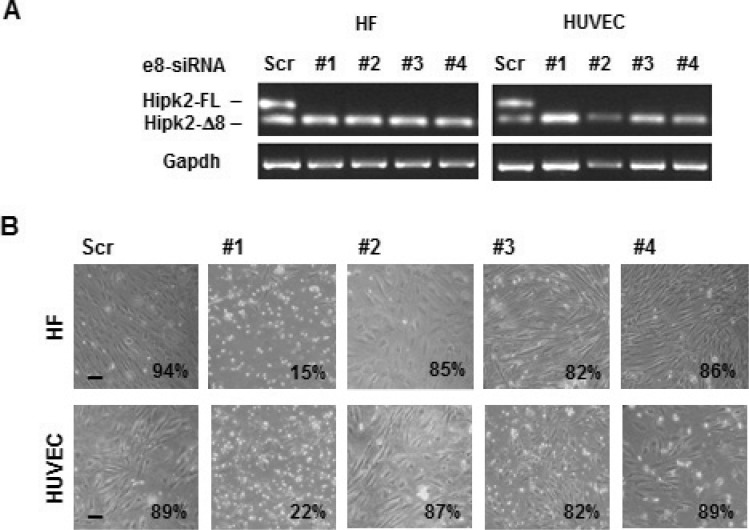
Non-tumor cell are sensitive to the apoptotic effect of e8-siRNA#1 Human immortalized fibroblast (HF) and primary endothelial cells (HUVEC) were transfected with the indicated siRNAs. (**A**) At 96 hours post-transfection viability was assessed and the Hipk2-FL depletion monitored by RT-PCR. (**B**) Light microscopy pictures of transfected cells taken 96 hrs post-transfection. The percentage of viable cells is reported at the right bottom of each panel. Bars, 100 μm.

Recognizing and avoiding siRNA off-target effects is mandatory to elucidate gene function. Interestingly, this absoluteness was shown to be less relevant for therapeutic applications [34]. In a last attempt to translate the strong reduction of cell viability induced by e8-siRNA#1 in cancer cells *in vitro* and *in vivo* into a therapeutic strategy for CRCs, we asked whether this treatment possesses a therapeutic window (*i.e*., normal cells are significantly less susceptible than cancer cells to the e8-siRNA#1-induced cell death). Off-target transcript regulation by siRNAs was shown to be species-specific and preclinical mouse models might not predict human off-target activity [35]. Thus, we analyzed the effect induced by e8-siRNA#1 and the other e8-specific siRNAs on the viability of non-tumor, primary human umbilical vein endothelial cells (HUVECs), and immortalized human dermal fibroblasts (HFs). As observed with cancer cells, both HUVECs and HFs showed clear and comparable repression of the Hipk2-FL isoform with all the employed e8-specific siRNAs and significant loss of viability only with the e8-siRNA#1 (Figure 6). These results show that the off-target-dependent cell death induced by e8-siRNA#1 is comparable between cancer and “normal” cells showing on one side, a broad consistency of this result, and, on the other side, the absence of a therapeutic window that strongly discourages further preclinical testing.

## DISCUSSION

In this study, we investigated whether the prevalence of HIPK2-Δe8 isoform induced by HIPK2 FL-specific siRNA can have therapeutic effects in CRC cells.

HIPK2 is an effective player in cell response to genotoxic agents that can sense damage intensity and involved in the choice between cell cycle arrest and apoptosis in p53-dependent and –independent manners [36]. Besides posttranslational modifications and subcellular localization, HIPK2 activity is tightly controlled by the ubiquitin–proteasome system and multiple HIPK2-targeting E3 ligases have been characterized [37]. Recently, two alternative splicing forms of HIPK2 with different sensitivity to the E3 ubiquitin ligase Siah-1 have been described, the Hipk2-FL and the Hipk2-Δe8 isoform, that lacks the 27 aminoacids required for Siah-1-binding, thus becoming resistant to proteasome digestion [27]. It is important to note that, by employing a specific siRNA that selectively depletes Hipk2-FL, a strong induction of apoptosis was observed in CRC cell lines highlighting a potential therapeutic strategy for CRCs, even against mutant p53-carrying tumors [27]. We took up the challenge of testing this hypothesis on patient-derived colorectal cancer stem cells, whose profiles have been shown to be highly prognostic for CRC patients [38]. We were able to confirm the existence of the two alternative splicing forms of Hipk2 in these CSCs as well as in cells from tumor lines or primary normal HUVEC and immortalized HFs (Figures 1 and 6). In addition, transfection with the originally employed Hipk2-FL-specific e8-siRNA#1 induces the reported imbalance between Hipk2-FL and the Hipk2-Δe8 isoforms that is associated with loss of viability both *in vitro* and *in vivo* (Figures 2, 3, and 6). Thus, we could easily reproduce the results obtained by Kurokawa and coworkers [27] in all the cells in which we performed these experiments.

The on-target specificity of the Hipk2-FL-specific e8-siRNA was originally assessed at the gene-expression level by employing two individual siRNAs targeting the skipped exon 8 region of Hipk2 and demonstrating a comparable imbalance between Hipk2 isoforms [27]. However, at the phenotypic level, *i.e.* the apoptotic outcome, only a single siRNA, the HIPK2 siRNA#2 (here named e8-siRNA#1) was employed [27], making the exclusion of possible off-target effects unreliable. Indeed, when we attempted to validate the target-specificity of e8-siRNA#1 by employing three additional e8-specific siRNAs, we also found that each siRNA was able to knockdown the expression of Hipk2-FL sparing the Hipk2-Δe8 isoforms. However, only the originally described e8-siRNA#1 reduces cell viability indicating that its apoptotic activity is caused by off-target effects (Figure 4). In addition, as demonstrated by experiments performed in Hipk2-KO cells, the e8-siRNA#1 strongly reduces cell viability even in the absence of HIPK2. This further reinforces the concept that the apoptotic effect is not caused by an unbalance between the two isoforms but is, indeed, HIPK2-independent. Thought less intriguing, this conclusion is coherent with the observation originally made by Kurokawa [27] and confirmed by us, here, showing that the two isoforms have similar activity upon their overexpression.

A growing body of evidence demonstrates that siRNA specificity is not absolute and off-target gene silencing can occur through different mechanisms, such as mRNA degradation mediated by partial sequence complementation, immune stimulation by interferon response, microRNA-like inhibition of translation, and saturation of the RNAi machinery [14]. In an attempt to characterize the off-target activity of the e8-siRNA#1, we observed that its lack of specificity is not due to partial sequence complementation and subsequent off-target mRNA degradation (Figure 5). Since loss of viability induced by e8-siRNA#1 was present both *in vitro* and *in vivo* in cells of non-immune origin (Figures 2, 3, and 6), we can exclude an immune stimulation-based off-target activity. The e8-siRNA#1 employed in the original work by Kurokawa [27] and by our group in our *in vitro* and *in vivo* experiments are chemically modified Stealth RNAi™ produced by Invitrogen. This type of modification, which is a company property, provides increased stability. Thus, we directly tested and experimentally ruled out the possibility that such stability might be responsible for off-target-induced cell death by saturation of the RNAi machinery, a further mechanism of off-target gene silencing [39]. By exclusion, at least to our current knowledge of the phenomenon, the remaining mechanism that may explain the e8-siRNA#1 off-target effect is the microRNA-like activity. siRNAs have been shown to repress subsets of genes by acting like microRNAs and inducing, in addition to the cleavage of the intended target, the translational repression of several unintended targets [40]. While the first effect is the result of a perfect base-pairing, the second is due to the sequence-dependent interaction of siRNA with non-intended targets through a few nucleotide sequence of the 5′ end of the siRNA guide strand. Our approach towards target validation also supports the hypothesis that the e8-siRNA#1 induces apoptosis by microRNA-like off-target activity. Indeed, the three further individual siRNAs we employed to target the same Hipk2-FL gene partially overlaps the original e8-siRNA sequence. The small slide on the gene sequence of the additional siRNAs (Figure 4A) allowed the maintenance of the Hipk2-FL specific repression but was sufficient to avoid cell-death, showing that a few nucleotide differences are sufficient to overcome the off-target activity.

Further analyses, such as RNA deep-sequencing, would have proven the hypothesis that e8-siRNA#1 induces cell death by microRNA-like activity. This type of characterization would have been essential in the case of a therapeutic application for CRC treatment. Indeed, it has been proposed that the need in recognizing and avoiding siRNA off-target effects is mandatory in the process of elucidating gene function but might be less relevant for therapeutic applications and some “undesirable” off-target effects might even become useful in medical strategy [34]. We took this option into consideration and began assessing the therapeutic window of the e8-siRNA#1-treatment by comparable siRNA transfection of cancer and “normal” human cells. Disappointingly for the translational approach, we have found comparable evidence of loss of viability in both cell types strongly persuading against further analyses. Conversely, these observations highlight the broad consistency of the e8-siRNA#1 off-target activity, claiming, once more, for the need for validating depletion experiments with multiple tools and assessing the consistency of both molecular and functional readouts.

## MATERIALS AND METHODS

### Cell culture and transfection

HCT116, H1299, U2OS cell lines and HF (immortalized Human Fibroblasts) were cultured in DMEM supplemented with 10% (v/v) FBS and antibiotics. HUVEC (Human Umbilical Cord Derived Endothelial cells) were cultured in EBM2 medium (Lonza). Hipk2-KO cells containing a frame shift mutation in Hipk2 exon 3 and their parental Hap1 cells were purchased from Horizon Genomics and cultured in IMDM medium supplemented with 10% FBS. To obtain CSCs, tumor samples were subjected to mechanical and enzymatic dissociation. The resulting cancer cells were cultured in a serum-free medium supplemented with 20 ng/ml EGF and 10 ng/ml FGF-2 (PrePro-Tech) [28]. Chemically modified Stealth RNAi (Invitrogen) or unmodified siRNAs (MWG Operon, Eurofin) were used for cell transfection with RNAi-MAX Lipofectamine (Invitrogen), according to manufacturer's instructions. BLOCK-iT Red Fluorescent oligo (Invitrogen) was used to measure RNA transfection efficiency. N-terminal GFP-tagged HIPK2-FL and HIPK2-Δe8 expression vectors were obtained by cloning human Hipk2 cDNAs into pEGFP-c2 (Clontech) and confirmed by direct sequencing. Cells were transfected by Lipofectamine LTX and Plus reagent (Invitrogen).

### RT–PCR

Total RNA was isolated using the RNeasy mini Kit (Qiagen). cDNA was synthesized from using a M-MLTV RTase and amplified with GoTaq DNA polymerase (Promega) Primer sequences are as follows:

Hipk2-FW GGCTGACCGGCGGGAGTT,
Hipk2-REV GGTCAGGCCGGGCACAAATCT (NCBI Ref Seq NM_022740.4);
NR1D2-FW AGTGAGAAGCTAAATGCCCTCC,
Nr1D2 REV TATCAGGGAGATGCAGTCAACG (NCBI Ref Seq NM_001145425.1).

For quantitative PCR analysis, mRNA expression level was evaluated using Power SYBR Green PCR master mix with ABI Prism 7500HT Fast Real-Time PCR System Detector (Applied Biosystems). Gene expression was quantified using the 2-ΔΔCt methods. *Gapdh* was used as endogenous reference gene. Primers sequences (Ref Seq: NM_001113239, NM_022740) are as follows:

Hipk2-FL-FW: TGACCATGACCTTTAACAACCA
Hipk2-FL-REV: GCTAAGGAAATAGTGGCACTGG
Hipk2-Δ8-FW: TGACCACTGTCCACAACCAG CCCTCAG
Hipk2-Δ8-REV: TCGCACCGAGTAGCCAGCGTGC

### Western blotting

Whole-cell lysates were prepared using RIPA buffer containing a protease and phosphatase inhibitor mixture (Roche Applied Science). The extracted proteins were separated by SDS-PAGE and then transferred onto a nitrocellulose membrane (Bio-Rad). After blocking for 1 hr at room temperature with 5% non-fat milk (Bio-Rad), membranes were incubated overnight at 4°C with the indicated antibodies. Following incubation with an appropriate secondary antibody for 1 hr at room temperature, bound antibodies were detected with an ECL WB Detection System (GE Healthcare). Apoptosis was evaluated by measuring cleaved Caspase-3 (Cell Signaling Technology), PARP-1 and β-Catenin (Abcam) levels. β-actin (Santa Cruz Biotecnology) was used as a loading control.

### Animal maintenance and tumor xenografts

Six-week old *nu/nu* male mice were purchased from Charles River and housed with laboratory chow and water available *ad libitum*. All procedures involving mice were performed in compliance with our institutional animal care guidelines and following national and international directives (D.L. March 4, 2014, no. 26; directive 2010/63/EU of the European parliament and of the council; Guide for the Care and Use of Laboratory Animals, United States National Research Council, 2011). Tumor xenografts were established by injecting subcutaneously into the right flank of mice 1 × 10^6^ patient-derived CSC1 cells stably transfected with a luciferase reporter vector [31] mixed with Matrigel (BD Biosciences). Seven days post injection, when tumors reached about 40 mm^3^ and were detectable by bioluminescent imaging, animals were randomized into two groups (*n* = 6 in each group), one receiving e8siRNA#1 and the other receiving a control siRNA (Scrambled-siRNA). siRNAs (10 μg/mouse) were intratumorally injected as complexes with 5 μl of RNAi-Max Lipofectamine. The injection protocol was performed twice a week for two weeks.

Tumor dimensions were measured using a digital caliper and tumor volume was calculated before each intratumoral injection. Tumor growth was also monitored by bioluminescent imaging as previously described [31]. Data were expressed as photon/second/cm^2^/steradiant (p/s/cm^2^/sr) and the scale used in each experiment is reported in the corresponding figures. Luciferase imaging was performed twice a week. Fifteen days after the first treatment mice were sacrificed and tumors were explanted, photographed and weighed.

### Statistical analyses

For comparison between two independent groups, the Student's *t*-test was used.

## References

[1] Ferlay J, Shin HR, Bray F, Forman D, Mathers C, Parkin DM (2010). Estimates of worldwide burden of cancer in 2008: GLOBOCAN 2008. Int J Cancer.

[2] Brenner H, Kloor M, Pox CP (2014). Colorectal cancer. Lancet.

[3] Ipsaro JJ, Joshua-Tor L (2015). From guide to target: molecular insights into eukaryotic RNA-interference machinery. Nat Struct Mol Biol.

[4] Caplen NJ, Mousses S (2003). Short interfering RNA (siRNA)-mediated RNA interference (RNAi) in human cells. Ann N Y Acad Sci.

[5] Caplen NJ, Parrish S, Imani F, Fire A, Morgan RA (2001). Specific inhibition of gene expression by small double-stranded RNAs in invertebrate and vertebrate systems. Pnas.

[6] Obbard DJ, Gordon KH, Buck AH, Jiggins FM (2009). The evolution of RNAi as a defence against viruses and transposable elements. Philos Trans R Soc Lond B Biol Sci.

[7] Falschlehner C, Steinbrink S, Erdmann G, Boutros M (2010). High-throughput RNAi screening to dissect cellular pathways: a how-to guide. Biotechnol J.

[8] Caplen NJ (2003). RNAi as a gene therapy approach. Expert Opin Biol Ther.

[9] Burnett JC, Rossi JJ, Tiemann K (2011). Current progress of siRNA/shRNA therapeutics in clinical trials. Biotechnol J.

[10] Davidson BL, McCray PB (2011). Current prospects for RNA interference-based therapies. Nature Reviews Genetics.

[11] Deng Y, Wang CC, Choy KW, Du Q, Chen J, Wang Q Li L, Chung TK, Tang T (2014). Therapeutic potentials of gene silencing by RNA interference: principles, challenges, and new strategies. Gene.

[12] Gondi CS, Rao JS (2009). Concepts in *in vivo* siRNA delivery for cancer therapy. J Cell Physiol.

[13] Videira M, Arranja A, Rafael D, Gaspar R (2014). Preclinical development of siRNA therapeutics: towards the match between fundamental science and engineered systems. Nanomedicine.

[14] Jackson AL, Linsley PS (2010). Recognizing and avoiding siRNA off-target effects for target identification and therapeutic application. Nat Rev Drug Discov.

[15] Saul VV, de la Vega L, Milanovic M, Krüger M, Braun T, Fritz-Wolf K, Becker K, Schmitz ML (2013). HIPK2 kinase activity depends on cis-autophosphorylation of its activation loop. J Mol Cell Biol.

[16] Siepi F, Gatti V, Camerini S, Crescenzi M, Soddu S (2013). HIPK2 catalytic activity and subcellular localization are regulated by activation-loop Y354 autophosphorylation. Biochim Biophys Acta.

[17] Rinaldo C, Moncada A, Gradi A, Ciuffini L, D'Eliseo D, Siepi F, Prodosmo A, Giorgi A, Pierantoni GM, Trapasso F, Guarguaglini G, Bartolazzi A, Cundari E (2012). HIPK2 controls cytokinesis and prevents tetraploidization by phosphorylating histone H2B at the midbody. Mol Cell.

[18] Mao JH, Wu D, Kim IJ, Kang HC, Wei G, Climent J, Kumar A, Pelorosso FG, DelRosario R, Huang EJ, Balmain A (2012). Hipk2 cooperates with p53 to suppress γ-ray radiation-induced mouse thymic lymphoma. Oncogene.

[19] D'Orazi G, Rinaldo C, Soddu S (2012). Updates on HIPK2: a resourceful oncosuppressor for clearing cancer. J Exp Clin Cancer Res.

[20] Hofmann TG, Glas C, Bitomsky N (2013). HIPK2: A tumour suppressor that controls DNA damage-induced cell fate and cytokinesis. Bioessays.

[21] Valente D, Bossi G, Moncada A, Tornincasa M, Indelicato S, Piscuoglio S, Karamitopoulou ED, Bartolazzi A, Pierantoni GM, Fusco A, Soddu S, Rinaldo C (2015). HIPK2 deficiency causes chromosomal instability by cytokinesis failure and increases tumorigenicity. Oncotarget.

[22] D'Orazi G, Cecchinelli B, Bruno T, Manni I, Higashimoto Y, Saito S, Gostissa M, Coen S, Marchetti A, Del Sal G, Piaggio G, Fanciulli M, Appella E, Soddu S (2002). Homeodomain-interacting protein kinase-2 phosphorylates p53 at Ser 46 and mediates apoptosis. Nat Cell Biol.

[23] Hofmann TG, Möller A, Sirma H, Zentgraf H, Taya Y, Dröge W, Will H, Schmitz ML (2002). Regulation of p53 activity by its interaction with homeodomain-interacting protein kinase-2. Nat Cell Biol.

[24] Zhang Q, Yoshimatsu Y, Hildebrand J, Frisch SM, Goodman RH (2003). Homeodomain interacting protein kinase 2 promotes apoptosis by downregulating the transcriptional corepressor CtBP. Cell.

[25] Krieghoff-Henning E, Hofmann TG (2008). HIPK2 and cancer cell resistance to therapy. Future Oncol.

[26] Puca R, Nardinocchi L, Givol D, D'Orazi G (2010). Regulation of p53 activity by HIPK2: molecular mechanisms and therapeutical implications in human cancer cells. Oncogene.

[27] Kurokawa K, Akaike Y, Masuda K, Kuwano Y, Nishida K, Yamagishi N, Kajita K, Tanahashi T, Rokutan K (2014). Downregulation of serine/arginine-rich splicing factor 3 induces G1 cell cycle arrest and apoptosis in colon cancer cells. Oncogene.

[28] Ricci-Vitiani L, Lombardi DG, Pilozzi E, Biffoni M, Todaro M, Peschle C, De Maria R (2007). Identification and expansion of human colon-cancer-initiating cells. Nature.

[29] Cammareri P, Lombardo Y, Francipane MG, Bonventre S, Todaro M, Stassi G (2008). Isolation and culture of colon cancer stem cells. Methods Cell Biol.

[30] Steinhusen U, Badock V, Bauer A, Behrens J, Wittman-Liebold B, Dörken B, Bommert K (2000). Apoptosis-induced Cleavage of β-Catenin by Caspase-3 Results in Proteolytic Fragments with Reduced Transactivation Potential. J Biol Chem.

[31] Di Rocco G, Gentile A, Antonini A, Truffa S, Piaggio G, Capogrossi MC, Toietta G (2012). Analysis of biodistribution and engraftment into the liver of genetically modified mesenchymal stromal cells derived from adipose tissue. Cell Transplant.

[32] Jackson AL, Linsley PS (2010). Recognizing and avoiding siRNA off-target effects for target identification and therapeutic application. Nat Rev Drug Discov.

[33] Burris TP (2008). Nuclear hormone receptors for heme: REV-ERBalpha and REV-ERBbeta are ligand-regulated components of the mammalian clock. Mol Endocrinol.

[34] Lares MR, Rossi JJ, Ouellet DL (2010). RNAi and small interfering RNAs in human disease therapeutic applications. Trends Biotechnol.

[35] Burchard J, Jackson AL, Malkov V, Needham RH, Tan Y, Bartz SR, Dai H, Sachs AB, Linsley PS (2009). MicroRNA-like off-target transcript regulation by siRNAs is species specific. RNA.

[36] Rinaldo C, Prodosmo A, Siepi F, Soddu S (2007). HIPK2: a multitalented partner for transcription factors in DNA damage response and development. Biochem Cell Biol.

[37] Saul VV, Schmitz ML (2013). Posttranslational modifications regulate HIPK2, a driver of proliferative diseases. J Mol Med (Berl).

[38] Zeuner A, Todaro M, Stassi G, De Maria R (2014). Colorectal cancer stem cells: from the crypt to the clinic. Cell Stem Cell.

[39] Khan AA, Betel D, Miller ML, Sander C, Leslie CS, Marks DS (2009). Transfection of small RNAs globally perturbs gene regulation by endogenous microRNAs. Nat Biotechnol.

[40] Scacheri PC, Rozenblatt-Rosen O, Caplen NJ, Wolfsberg TG, Umayam L, Lee JC, Hughes CM, Shanmugam KS, Bhattacharjee A, Meyerson M, Collins FS (2004). Short interfering RNAs can induce unexpected and divergent changes in the levels of untargeted proteins in mammalian cells. Proc Natl Acad Sci. U S A.

